# Therapeutic potential of iron oxide nanoparticles for cutaneous leishmaniasis: a systematic review of *in vitro* and *in viv*o studies

**DOI:** 10.1590/1678-9199-JVATITD-2025-0004

**Published:** 2025-10-03

**Authors:** Priscila de Cássia da Silva, Bruna de Macedo Lima, Camila Sales Nascimento, Anna Carolina Pinheiro Lage, Celso Pinto de Melo, Carlos Eduardo Calzavara-Silva, Érica Alessandra Rocha Alves

**Affiliations:** 1 Cellular and Molecular Immunology Research Group, René Rachou Institute, Oswaldo Cruz Foundation (Fiocruz Minas), Belo Horizonte, MG, Brazil.; 2 Biotechnology Applied to the Study of Pathogens Research Group, René Rachou Institute, Oswaldo Cruz Foundation (Fiocruz Minas), Belo Horizonte, MG, Brazil.; 3 Non-Conventional Polymers Group, Department of Physics, Federal University of Pernambuco, Recife, PE, Brazil.

**Keywords:** Nanoparticles, Iron oxide, Cutaneous leishmaniasis, Leishmania, Antiprotozoal agents, Nanomedicine, Systematic review

## Abstract

The treatment of cutaneous leishmaniasis (CL) is challenged by limited therapeutic options, high drug toxicity, and frequent treatment failure. In this context, iron oxide nanoparticles (IONPs) have emerged as promising therapeutic alternatives. This review summarizes experimental findings on the *in vitro* and *in vivo* anti-*Leishmania* activity of IONPs, highlighting their potential as a treatment for CL. A systematic search of PubMed, ScienceDirect, and Scopus identified 16 studies evaluating the anti-*Leishmania* effects of IONPs across various CL models. The studies assessed IONPs' physicochemical properties (size, shape, polydispersity index, and zeta potential), functionalization strategies, and efficacy against axenic and intracellular *Leishmania* forms, as well as in animal models. Most studies investigated spherical IONPs ranging from 5 to 90 nm, with polydispersity index values between 0.2 and 1.0 and zeta potentials from -13 mV to +35 mV. Functionalization improved dispersion and enabled antimicrobial conjugation. IONPs reduced axenic *Leishmania* viability, decreased intracellular parasitism, and lowered parasite loads in infected mouse lesions. *In vitro*, parasite death was linked to lysosomal rupture, oxidative stress, apoptosis, necrosis, and nitric oxide production by macrophages. *In vivo*, treated animals exhibited reduced parasite burdens, milder lesions, and enhanced IFN-γ production, suggesting improved immune responses. Despite these promising effects, issues such as formulation optimization, biocompatibility, and evaluation of pharmacokinetics and pharmacodynamics remain to be addressed. IONPs represent a novel and promising dual-action therapeutic strategy for CL, combining antiparasitic effects with immune modulation. However, important knowledge gaps persist regarding their mechanisms of action, long-term safety, efficacy across different *Leishmania* species and clinical scenarios. Further research is needed to advance IONPs as a safe and effective treatment for CL.

## Graphical abstract

**Figure:**
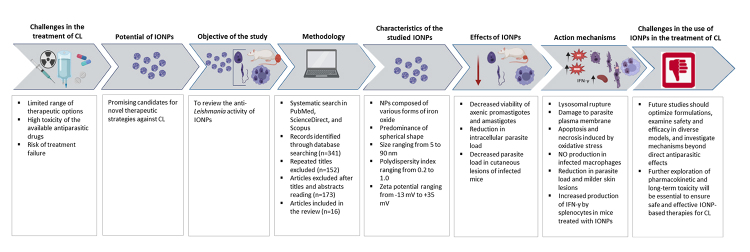
Graphical abstract

## Background

Cutaneous leishmaniasis (CL) is a neglected disease present in over 90 countries, with endemic transmission mainly in tropical and subtropical regions, including rural areas, tropical forests, arid zones, as well as semi-urban and urban settings [1]. The World Health Organization (WHO) estimates that between 600,000 and 1,000,000 new cases occur annually worldwide [2 **,** 3], though 70-75% of these cases are concentrated in countries such as Afghanistan, Algeria, Brazil, Colombia, Iran, Pakistan, Peru, Saudi Arabia, and Syria [2 **,** 4]. Human infection happens when infected sandflies inject metacyclic promastigotes into the skin during a blood meal. These promastigotes are engulfed by macrophages, where they differentiate into amastigotes within phagolysosomes, causing CL [5 **,** 6 **,** 7]. The disease primarily affects the skin and can have severe psychological consequences, including reduced quality of life, social stigma, and decreased self-esteem, depending on lesion severity, type, and scarring [8].

Multiple *Leishmania* species cause CL in humans. In the Eastern Hemisphere, the main species include *L. (L.) tropica*, *L. (L.) major*, and *L. (L.) aethiopica*. In the Americas, species such as *L. (L.) mexicana*, *L. (L.) amazonensis*, *L. (L.) venezuelensis*, *L. (V.) braziliensis*, *L. (V.) shawi*, *L. (V.) guyanensis*, *L. (V.) panamensis*, *L. (V.) peruviana*, *L. (V.) lainsoni*, *L. (V.) naiffi*, and *L. (V.) lindenberg* are involved [1]. Additionally, atypical cases caused by *L. infantum* have been reported in the Mediterranean and in Central and South America [4]. CL may be presented as localized or disseminated lesions affecting multiple body areas. In some cases, the cutaneous form can also progress to mucosal involvement. Localized disease is generally less severe and typically responds well to treatment. In contrast, disseminated and mucosal forms tend to be more resistant to therapy, often requiring multiple or specialized treatments to achieve cure [4 **,** 9 **,** 10 **,** 11]. The clinical manifestations depend on a complex interplay of factors, including the *Leishmania* species, host genetics, the type of immune response, and the presence of comorbidities [11 **,** 12]. These factors influence both disease severity and treatment response.

WHO recommends pentavalent antimonials (Sb^5+^) as the first-line treatment for CL. These include meglumine antimoniate (MA) and sodium stibogluconate (SSG), marketed as Glucantime® and Pentostam®, respectively [13 **,** 14]. These drugs can be administered intramuscularly, intravenously, or intralesionally. Intralesional administration reduces systemic side effects but is limited to localized disease forms [14 **,** 15 **,** 16]. Amphotericin B, available as deoxycholate or liposomal formulations and given intravenously, is a second-line treatment, particularly for mucosal and localized lesions unresponsive to antimonials [17 **,** 18]. Pentamidine, available as isethionate and mesylate forms for intramuscular injection, is also used as a second-line option in endemic regions across the Americas, Asia, and Africa [14 **,** 19 **,** 20]. In Brazil, miltefosine - initially approved for cancer treatment - has recently been added as a second-choice drug for patients who fail antimonials or for whom systemic antimony is contraindicated [13 **,** 14 **,** 21].

Although effective, these drugs have significant adverse effects. Common side effects include nausea, vomiting, weakness, myalgia, abdominal cramps, diarrhea, and skin rashes [22 **,** 23]. For intralesional MA, local reactions such as pain, edema, erythema, and pruritus are common [13 **,** 16 **,** 24]. More serious reactions include thrombophlebitis, nephrotoxicity, hypokalemia, myocarditis, hepatic failure, and acute pancreatitis. Miltefosine is teratogenic, requiring women of childbearing age to avoid pregnancy during treatment and for three months afterward [13 **,** 14 **,** 16 **,** 18 **,** 20 **,** 25 **,** 26].

Therapeutic failure or suboptimal responses also occur due to factors like reduced drug accumulation in parasites, host immunosuppression (from co-infections or malnutrition), and environmental conditions favoring resistant parasites [23 **,** 27 **,** 28]. In Colombia, treatment failure occurred in 15.65% of CL cases, with miltefosine showing a lower failure rate (8.92%) than MA (22.03%). Factors related to failure included age ≤ 8 years, disease duration ≤ 1-month, regional lymphadenopathy, and treatment adherence < 90% [29]. In French Guiana, pentamidine isethionate failure was higher with intramuscular administration (48.7%) *versus* intravenous (14.7%) [29]. In Mato Grosso, Brazil, a 47% failure rate with MA was reported, especially at lower doses, with risk factors including prior treatment, multiple lesions, irregular administration, and patient weight over 68 kg [30]. Amphotericin B treatment failures have also been documented in CL caused by *L. (V.) braziliensis* [31]. These findings underscore the challenges associated with treating CL and the urgent need to develop new therapeutic alternatives for this significant and neglected parasitic disease.

Nanotechnology has enabled the development of various therapeutic systems targeting protozoan diseases, primarily serving as carriers for antiparasitic drugs [32]. Leishmaniasis, in particular, is a compelling candidate for treatment with nanocarriers because the parasites infect highly phagocytic cells capable of internalizing drug-loaded nanoparticles, enhancing the elimination of intracellular pathogens [33 **,** 34]. Studies have demonstrated that nanoparticles carrying anti-*Leishmania* drugs are engulfed by macrophages, which then release the drugs into phagolysosomes containing amastigotes. This approach improves the drugs’ antiparasitic efficacy while minimizing side effects and toxicity [19 **,** 34 **,** 35].

Nanoparticles (NPs) are particles sized between 1 and 100 nm, granting them a high surface-to-volume ratio and unique physicochemical properties. They typically consist of a core surrounded by an outer layer, which can be functionalized with small molecules, metal ions, surfactants, or polymers to modify their characteristics [34 **,** 36 **,** 37]. Their small size enables entry into target cells through phagocytosis, macropinocytosis, or endocytosis [38]. These features make CL particularly suitable for nanoparticle-based treatments, as the parasites infect highly phagocytic cells that readily internalize NPs, facilitating their delivery into the intracellular environment and promoting parasite destruction [33].

In CL, both organic nanoparticles - such as liposomes, nanoemulsions, lipid nanoparticles, and carbon-based nanomaterials - and inorganic nanoparticles, especially metal and metal oxide nanoparticles, have been studied extensively [33, 32, 39]. Among metal oxide NPs, iron oxide nanoparticles (IONPs) have gained attention as promising therapeutic agents. Iron oxide is abundant in nature and exists in several forms with distinct magnetic properties, including magnetite (Fe_3_O_4_), maghemite (γ-Fe_2_O_3_), and hematite (α-Fe_2_O_3_), which are the most studied for medical use [40, 41].

Various laboratory techniques have been developed to produce iron oxide nanoparticles, such as co-precipitation, sol-gel synthesis, microemulsion, and thermal decomposition [40, 42, 43]. IONPs are notable for their biocompatibility, owing to iron’s natural occurrence in the body, contributing to their physiological stability and low toxicity. Moreover, synthesis methods can be tailored to achieve specific compositions and morphologies, with overall production processes being relatively simple and cost-effective [40, 43, 44]. This review covers different types of IONPs investigated for anti-*Leishmania* therapy, discussing their synthesis, biocompatibility, and applications, while also addressing existing gaps in knowledge regarding their therapeutic potential in treating CL.

## Methods

### Eligibility criteria

Eligible articles were those investigating the leishmanicidal effects of IONPs against species responsible for cutaneous leishmaniasis. To qualify for inclusion, studies needed to examine the effects of IONPs on axenic promastigotes and amastigotes, as well as intracellular amastigotes. Additionally, *in vivo* therapy studies were included, whether conducted independently or as part of the same research. Only original research articles published in the last ten years and written in English were considered, while reviews and letters to the editor were excluded.

### Sources of information and search strategy

All articles included in this review were identified through a systematic search of eligible publications in the ScienceDirect, PubMed, and Scopus databases. MeSH terms were not employed in the search strategy; instead, a combination of keywords - including synonyms and variations of key terms - was used. This approach allowed for a broader search scope compared to using MeSH terms, thereby increasing the likelihood of identifying a wider range of relevant articles. The selected keywords and their combinations were consistently applied across all three databases.

The exact Boolean operators used in the search were as follows: (“iron oxide nanoparticles” AND “leishmaniasis”), (“iron oxide nanoparticles” AND “cutaneous leishmaniasis”), (“iron oxide nanoparticles” AND “antileishmanial”), and (“iron oxide nanoparticles” AND “leishmaniasis treatment”). Additional searches included combinations such as (“magnetite nanoparticles” AND “cutaneous leishmaniasis”), (“maghemite nanoparticles” AND “cutaneous leishmaniasis”), and (“hematite nanoparticles” AND “cutaneous leishmaniasis”).

**Figure 1.**
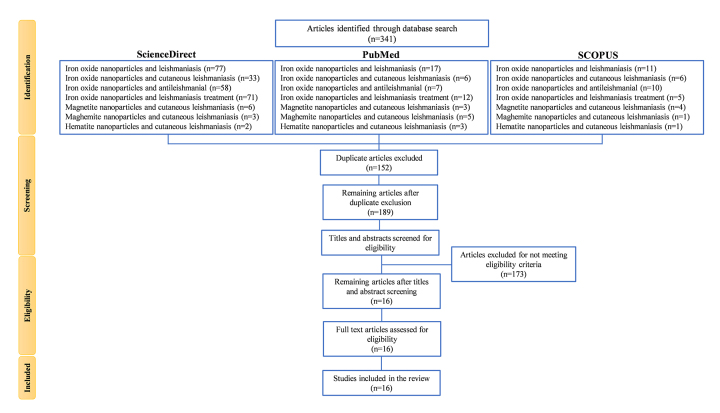
Flowchart summarizing the search strategy and selection process for articles included in the systematic review. Relevant studies published between January 2014 and December 2024 were initially identified using standardized keyword combinations across the ScienceDirect, PubMed, and Scopus databases. Retrieved articles were compiled manually in an Excel spreadsheet, and duplicate entries were removed. The titles and abstracts of the remaining records were screened to assess compliance with the eligibility criteria. Studies that were not original research or not published in English were excluded. The full texts of the eligible articles were then reviewed, and those meeting all predefined inclusion criteria were incorporated into the final analysis.

A publication date filter was applied to include studies published between January 2014 and December 2024. Titles and abstracts of the retrieved articles were manually compiled into an Excel spreadsheet, and duplicate entries were removed. The remaining titles and abstracts were then screened to identify articles that met the predefined eligibility criteria. Following this initial screening, the full texts of potentially eligible articles were accessed via database download links. Although not all articles were available for download, full access was obtained through our institution’s subscription. The full texts were then reviewed in detail to exclude any studies that did not meet the inclusion criteria. Ultimately, all articles that satisfied the eligibility requirements were included in the final review. A comprehensive flowchart outlining the search strategy is presented in Figure 1.

The data extracted from the included articles were manually compiled into tables using Word and Excel. The entire process of article search, identification, full-text review, and data extraction was independently conducted by two reviewers. Any discrepancies were resolved through discussion.

## Results

### Anti-*Leishmania* activity of iron oxide nanoparticles against axenic forms without concurrent biocompatibility assessment

**Figure 2.**
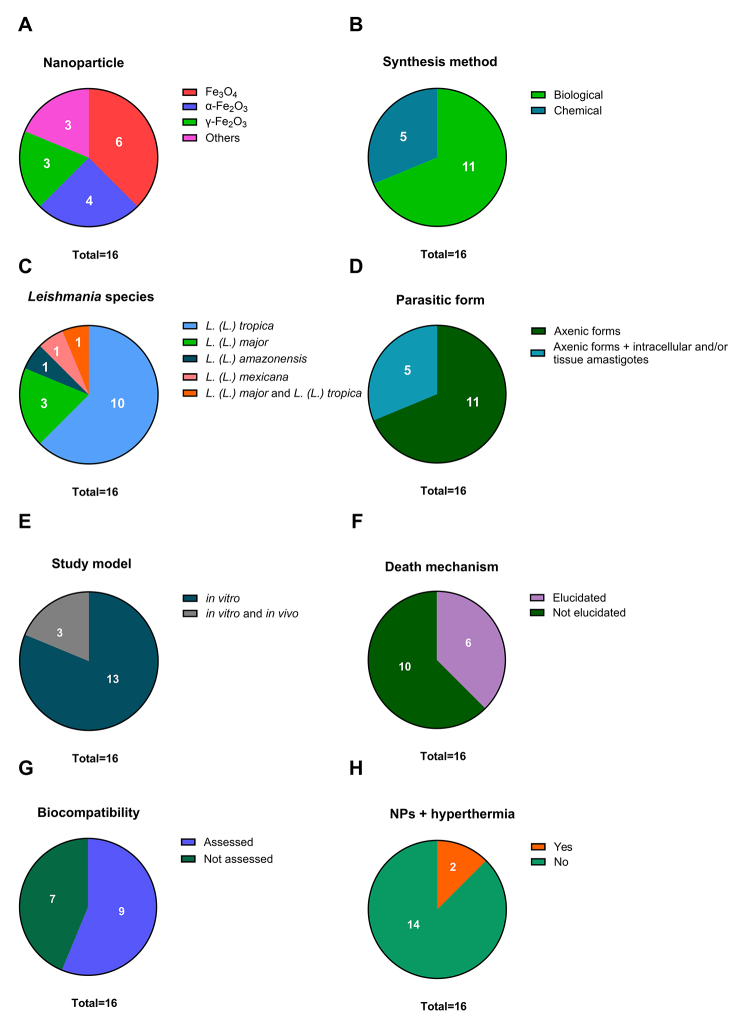
Profile of the sixteen articles included in the review. The included studies are categorized by type of **(A)** nanoparticle, **(B)** synthesis method, **(C)** *Leishmania* species, **(D)** parasitic form, **(E)** study model, **(F)** mechanism of parasite death, **(G)** biocompatibility assessment, and **(H)** the use of iron oxide nanoparticles combined with hyperthermia treatment.

As shown in Figure 2, out of sixteen articles published in the past decade investigating the anti-*Leishmania* activity of iron oxide nanoparticles (IONPs), sixteen specifically examined their effects on axenic forms of *Leishmania*. These studies consistently demonstrated significant reductions in parasite viability, as summarized in Table 1.

**Table 1.** Studies investigating the impact of iron oxide nanoparticles (IONPs) in various models of cutaneous leishmaniasis.

Nanoparticle	Morphological and dispersion characteristics	Species	Parasitic form	NPs effects	Death mechanism	Biocompatibility	Ref.
Fe_3_O_4_ NPs biosynthesized using *Rosmarinus officinalis* (rosemary) extract	Spherical shape, size ~ 5 nm and monodisperse	*L. (L.) major*	Promastigotes	***In vitro*:** After 72h ( viability, with an IC_50_ of 350 µg/mL ***In vivo*:** NA	NA	NA	[35]
Fe₃O₄ SPIONs synthesized by chemical method	Spherical shape, size ≥ 8 nm, and polydispersity index = 0.199	*L. (L.) tropica*	Promastigotes	***In vitro*:** After 72h ( growth with 23,2 µg/mL resulting in 70% inhibition ***In vivo*:** NA	NA	NA	[45]
FeO NPs biosynthesized using extract from *Anthemis tomentosa* flower	Spherical shape, size from 60 to 90 nm, and high dispersion	*L. (L.) tropica*	Promastigotes	***In vitro*:** After 72h ( viability, with an IC_50_ of 80.7 µg/mL ***In vivo*:** NA	NA	NA	[46]
α-Fe₂O₃ NPs biosynthesized using an aqueous extract from *Rhus punjabensis*	Rhombohedral shape and size ~ 41.0 ± 5 nm	*L. (L.) tropica*	Axenic amastigotes	***In vitro*:** After 24h ( viability, with an IC_50_ of 20 µg/mL ***In vivo*:** NA	NA	NA	[47]
α-Fe₂O₃ NPs biosynthesized using aqueous extracts from *Annona squamosa* peels	Spherical shape and size from 20 to 33 nm	*L. (L.) tropica*	Promastigotes	***In vitro*:** After 96h ( growth inhibition from 52.0% to 86.6% when using the 4:1 ratio ***In vivo*:** NA	NA	NA	[48]
MAA)-functionalized Fe₃O₄@bio-MOFs nanocomposites synthesized by chemical method	MAA-functionalized Fe₃O₄ NPs: spherical shape, size ~ 35 nm, and monodisperse; bio-MOFs: open, porous layers, smooth surface, homogeneously dispersed, thickness from 30 to 60 nm	*L. (L.) major*	Promastigotes ( Intracellular amastigotes ( Amastigotes in tissues	***In vitro*:** After 72h ( viability, with an IC_50_ of 12.5 ± 0.47 µg/mL. Also, ( intracellular amastigotes. In the control group the average number of parasites per cell was 5.5, while in the group treated with 6,25, 25.0, 50.0 and 100 µg/mL was 2.8, 1.9, 1.4 and 0.95, respectively ***In vivo*:** After topical treatment**,** ( lesions and parasite load in the skin of infected BALB/c mice	***In vitro*:** NA ***In vivo*:** ( IFN-γ by T lymphocytes	Toxic to murine macrophages at concentrations of 12.5 µg/mL and higher	[49]
γ-Fe₂O₃ NPs biosynthesized using an aqueous extract from *Sageretia thea* (Osbeck)	Tetragonal shape and size ~ 29 nm	*L. (L.) tropica*	Promastigotes Axenic amastigotes	***In vitro*:** After 24h ( viability, with an IC_50_ of 17,2 µg/mL e 16,75 µg/mL, for promastigotes and amastigotes, respectively ***In vivo*:** NA	NA	Toxic to human erythrocytes at moderate or high concentrations Non-toxic to human macrophages (IC_50_ ˃ 200 µg/mL)	[50]
α-Fe₂O₃ NPs biosynthesized using extract from *Callistemon viminalis* flowers	Spherical and size from 22 to 32 nm	*L. (L.) tropica*	Promastigotes Axenic amastigotes	***In vitro*:** After 72h ( viability, with an IC_50_ of 40.8 µg/mL for promastigotes and 56.28 µg/mL for amastigotes, respectively ***In vivo*:** NA	NA	Toxic to human erythrocytes only at higher concentrations (1000 µg/mL)	[51]
α-Fe₂O₃ NPs biosynthesized using aqueous extract from *Rhamnus virgata* (Roxb) leaves	Spherical shape, size ~ 20 nm, polydispersity index = 1.0, and ζ potential = −13mV	*L. (L.) tropica*	Promastigotes Axenic amastigotes	***In vitro*:** After 72 h ( viability, with an IC_50_ of 8.08 µg/mL for promastigotes and 20.6 µg/mL for amastigotes ***In vivo*:** NA	NA	Non-toxic to human erythrocytes at low concentration (2 µg/mL) ( Toxic to human macrophages at high concentration (200 µg/mL)	[52]
PEI_25_-CAN-γ-Fe₂O₃ NPs synthesized by chemical method	Spherical shape, size from 7 to 15 nm, polydispersity index = 0.18- 0.207, and ζ potential = +25-35 mV	*L. (L.) major L. (L.) tropica*	Promastigotes Amastigotes in tissues	***In vitro*:** After 24h ( viability for promastigotes of both species ***In vivo*:** After topical treatment**,** ( skin of infected BALB/c mice	***In vitro*:** Cytolysis caused by the rupture of the single lysosome of the *Leishmania* parasites ***In vivo*:** NA	Toxic to THP-1 macrophages on at concentration 1,5 µg/mL	[53]
-Fe₂O₃ NPs biosynthesized using *Rhamnella gilgitica* leaf extract	Spherical shape, size ~ 20 nm, polydispersity index = 0.737, and ζ potential = -8.7 mV	*L.(L) tropica*	Promastigotes Axenic amastigotes	NA	Non-toxic to human cells	[54]
Fe_3_O_4_ SPIONs biosynthesized using coconut water	Spherical shape, size ~ 4.0 nm, and ζ potential = -8.7 mV	*L. (L.) amazonensis*	Promastigotes Intracellular amastigotes	***In vitro:*** Only ( viability of intracellular amastigotes ***In vivo*:** NA	***In vitro*:** Amastigotes showed lipid bodies, cytoplasmic disorganization with autophagic vacuoles, myelin-like figures, and mitochondrial swelling ***In vivo:*** NA	Non-toxic to RAW cells	[55]
FeO NPs biosynthesized using extract from *Leptolyngbya* sp. L-2	Spherical shape, size ~ 23 nm, polydispersity index 0.761, and ζ potential -8.5 mV.	*L. (L.) tropica*	Promastigotes Axenic amastigotes	***In vitro***: After 72h ( viability of both axenic forms, with an IC_50_ of 10,73 µg/mL for promastigotes and 16,89 µg/mL for amastigotes ***In vivo*:** NA	NA	Non-toxic to human cells	[56]
FIONs biosynthesized using an aqueous extract of *Trigonella foenum-graecum* (fenugreek) seed	Polyhedral shape, size ~ 70 nm, polydispersity index *<*0.1, and ζ potential -23.4 ± 5.6	*L. (L.) tropica*	Promastigotes Intracellular amastigotes	***In vitro***: Exposure to FIONs combined with LED light of 84 Im/W ( viability of promastigotes, with an IC_50_ of 0.036 ± 0.003 μg/mL and ( intracellular parasite load in peritoneal macrophages ***In vivo*:** NA	***In vitro***: Apoptosis and necrosis induced by oxidative stress ***In vivo***: NA	Non-toxic to human erythrocytes and mouse peritoneal macrophages	[57]
CA-coated Fe₃O₄ NPs, produced by chemical method	Spherical shape, size ≥ 55nm, polydispersity index = 0.20, and ζ potential -51.70 mV	*L. (L.) mexicana*	Axenic amastigotes	***In vitro***: Exposure of Fe₃O₄ NPs to an alternating magnetic field of 30 mT at a frequency of 452 kHz for 40 min ( viability of amastigotas *In vivo:* NA	***In vitro:*** Damage to the cytoskeleton and cellular architecture. ***In vivo:*** NA	NA	[58]
PO-coated Fe₃O₄ NPs synthesized by chemical method	Spherical shape and size from 15 to 20 nm	*L. (L.) major*	Promastigotes Intracellular amastigotes Amastigotes in tissues	***In vitro***: After 2 days, ( parasite load of macrophages, with and IC_50_ of 31,3 ± 2,26 and 62,3 ± 2,15 μg/mL for Fe_3_O_4_ NPs coated or not with PO, respectively. ***In vivo:*** After topical treatment, ( lesions and parasite load in the skin of infected BALB/c mice	***In vitro***: NO production by macrophages, damage to the parasite’s plasma membrane ***In vivo*:** NA	NA	[59]

In one study, magnetite (Fe_3_O_4_) NPs were biosynthesized using *Rosmarinus officinalis* (rosemary) leaf extract. Characterization revealed spherical particles averaging 5 nm in size with a uniform distribution. The NPs were tested against *L. (L.) major* promastigotes, showing a significant reduction in viability. After 72 hours of incubation with Fe₃O₄ NPs at concentrations between 1.0 and 400 µg/mL, the IC_50_ was determined to be 350 µg/mL [35].

Similarly, Fe_3_O_4_ superparamagnetic iron oxide nanoparticles (SPIONs) produced via a conventional chemical synthesis exhibited a mainly spherical morphology, averaging 8 nm in size and possessing a polydispersity index of 0.199, indicating moderate size heterogeneity. When incubated with *L. (L.) tropica* promastigotes at concentrations from 0.232 to 23.2 µg/mL over 72 hours, a dose-dependent reduction in parasite viability was observed, with the highest concentration achieving 70% inhibition of growth [45].

Iron oxide (FeO) NPs were biosynthesized using *Anthemis tomentosa* flower extract. These spherical particles ranged from 60 to 90 nm and were highly dispersed. After 72 hours of incubation with *L. (L.) tropica* promastigotes at concentrations between 15.12 and 2000 µg/mL, the number of viable parasites decreased, with an IC_50_ of 80.7 µg/mL. However, compared to meglumine antimoniate (MA), the reference treatment for CL, FeO NPs were less potent, as MA showed an IC_50_ of 5.11 µg/mL [46].

Hematite (α-Fe_2_O_3_) NPs biosynthesized using *Rhus punjabensis* aqueous extract exhibited a rhombohedral shape with an average size of 41 ± 5 nm. Their toxicity was evaluated against axenic amastigotes of *L. (L.) tropica*, compared to the plant extract alone. Parasites were incubated with different volumes of NPs for 24 hours at 25°C, and viability was assessed using the MTT assay. The α-Fe_2_O_3_ NPs showed significant anti-amastigote activity, with an IC_50_ of 20 µg/mL, substantially more effective than the extract, which had an IC_50_ of 101 µg/mL [47].

Additionally, α-Fe_2_O_3_ NPs biosynthesized from *Annona squamosa* peel extract had spherical morphology and sizes between 20 and 33 nm. In proliferation assays*, L. (L.) tropica* promastigotes were incubated with NPs at various ratios (1:1, 1:2, 2:1, 4:1) for up to 96 hours. The growth inhibition rate increased with higher NP concentrations, reaching 86.6% at the 4:1 ratio, demonstrating strong anti-*Leishmania* activity [48].

### Anti-*Leishmania* activity of iron oxide nanoparticles against axenic forms with simultaneous biocompatibility assessment

Although the previously mentioned studies highlight the promising anti-*Leishmania* properties of IONPs, none evaluated their toxicity in mammalian cells, including macrophages, which are the primary host cells of *Leishmania*. To address this limitation, some studies have assessed both anti-*Leishmania* activity and biocompatibility of IONPs in various cellular models. As shown in Figure 2, among the sixteen studies included in this review, nine investigated the biocompatibility of IONPs, mainly using macrophages and erythrocytes.

In one study, MAA-functionalized Fe_3_O_4_@bio-MOFs nanocomposites were synthesized using a conventional chemical method. Biological metal-organic frameworks (bio-MOFs) are hybrid structures that combine metal ions with organic ligands, forming porous materials with biomedical applications. In this case, MAA-functionalized Fe_3_O_4_ NPs were incorporated into bio-MOFs [49]. MAA-functionalized Fe₃O₄ NPs presented spherical shape, with a mean size of 35 nm and uniform distribution, while the bio-MOFs displayed smooth and porous layers between 30 and 60 nm thick. Promastigotes of *L. (L.) major* and J774 macrophages were exposed to MAA-functionalized Fe_3_O_4_@bio-MOFs (3.12-400 µg/mL, 72 h). A dose-dependent reduction in parasite viability was observed, with an IC_50_ of 12.5 ± 0.47 µg/mL. However, significant macrophage toxicity occurred at doses ≥12.5 µg/mL [49]. These findings suggest that although the nanocomposites are active against *Leishmania*, they may present potential risks to host cells.

Likewise, maghemite (γ-Fe_2_O_3_) NPs biosynthesized using aqueous extract of *Sageretia thea* (Osbeck) were evaluated for anti-*Leishmania* activity and biocompatibility. These NPs had a tetragonal shape and an average size of ~29 nm. When *L. (L.) tropica* promastigotes and amastigotes were exposed to the NPs (1-200 µg/mL, 24 h), IC_50_ values were 17.2 µg/mL and 16.75 µg/mL, respectively [50]. In human macrophages, cell viability decreased in a dose-dependent manner, with 47 ± 2.34% inhibition at 200 µg/mL and only 3.6 ± 1.1% at 1 µg/mL. The macrophage IC_50_ was > 200 µg/mL. Hemolysis occurred at concentrations ≥ 10 µg/mL, while concentrations of 1-5 µg/mL had minimal impact. These results suggest higher toxicity to parasites than to host cells, supporting the therapeutic potential of these IONPs.

Hematite (α-Fe_2_O_3_) NPs synthesized using floral extract of *Callistemon viminalis* also showed anti-*Leishmania* activity. The spherical particles, 22-32 nm in size, caused a concentration-dependent reduction in *L. (L.) tropica* promastigotes and axenic amastigotes. Exposure to concentrations ranging from 1 to 200 µg/mL for 72 h resulted in IC_50_ values of 40.8 µg/mL and 56.28 µg/mL, respectively [51]. Hemolysis assays (31.25-1000 µg/mL) showed 22 ± 1.3% hemolysis at 1000 µg/mL and only 2.3 ± 0.24% at 31.25 µg/mL. These findings indicate that α-Fe_2_O_3_ NPs exhibit potent anti-*Leishmania* activity and are hemocompatible at lower therapeutic concentrations.

Another study synthesized α-Fe_2_O_3_ NPs using aqueous extract of *Rhamnus virgata* (Roxb) leaves. These particles were ~20 nm in size, spherical, with a polydispersity index of 1.0 and a zeta potential of −13 mV, indicating a broad size distribution and a moderate probability of aggregation. When *L. (L.) tropica* promastigotes and amastigotes were treated with the NPs (1-200 µg/mL, 72 h), IC_50_ values were 8.08 µg/mL and 20.82 µg/mL, respectively [52]. No hemolysis was observed at concentrations ≤ 2 µg/mL, while macrophage viability decreased by 31% at 200 µg/mL, indicating cytotoxicity at high concentrations. Nevertheless, the NPs exhibited higher toxicity toward parasites than host cells, suggesting therapeutic utility.

**Figure 3.**
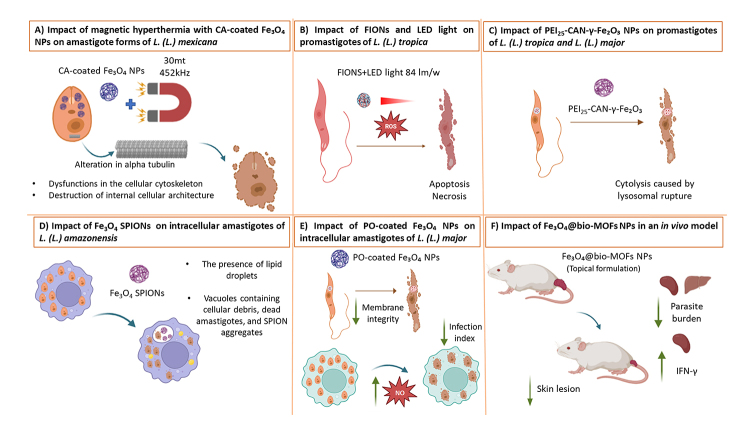
Hypothetical action mechanisms of iron oxide nanoparticles evaluated in the context of cutaneous leishmaniasis. **(A)** *L. (L.) mexicana* amastigotes treated with magnetic hyperthermia using CA-coated Fe_3_O_4_ nanoparticles. There were alterations in α-tubulin protein and destruction of cellular architecture, culminating in the reduction of parasite viability. **(B)** Exposure of *L. (L.) tropica* promastigotes to FIONS combined with light emitted by an LED lamp (84 lm/W) resulted in parasite death through apoptosis and necrosis induced by oxidative stress. **(C)** *L. (L.) tropica* and *L. (L.) major* promastigotes treated with PEI_25_-CAN-γ-Fe_2_O_3_ exhibited parasite death, attributed to cytolysis caused by the rupture of their single lysosome, induced by the presence of PEI. **(D)** Biosynthesized Fe_3_O_4_ SPIONs with coconut water significantly reduced the number of *L. (L.) amazonensis* amastigotes inside macrophages. Electron microscopy revealed alterations in treated amastigotes, such as lipid bodies, cytoplasmic disorganization, and vacuoles containing cellular debris. **(E)** Treatment of *L. (L.) major*-infected J774-A1 cells with PO-coated Fe_3_O_4_ nanoparticles for two days significantly reduced the macrophage infection index. This was accompanied by increased nitric oxide production by parasitized cells and compromised membrane integrity in the parasites. **(F)** Fe_3_O_4_@bio-MOFs (25 µg/mL) significantly reduced lesion size and parasite load in the spleen and liver of infected animals. After 21 days of treatment, there was a significant increase in IFN-γ production in splenic lymphocytes.

A distinct approach employed chemically synthesized γ-Fe_2_O_3_ NPs, which were doped with cerium (Ce³⁺/⁴⁺) using ceric ammonium nitrate (CAN) during synthesis. These nanoparticles were subsequently coated with polyethyleneimine (PEI), resulting in PEI_25_-CAN-γ-Fe_2_O_3_ NPs. TEM images revealed spherical particles of 7-15 nm, while DLS analysis indicated a polydispersity index of 0.18-0.207 and a zeta potential of +25-35 mV, indicating good dispersion and minimal aggregation [53]. These NPs were tested against *L. (L.) major* and *L. (L.) tropica* promastigotes at concentrations of 0.25 and 0.37 µg/mL for 24 h, showing potent activity attributed to PEI-induced lysosomal rupture (Figure 3). THP-1 macrophages treated with 0.5-2.0 µg/mL showed significant cytotoxicity only at concentrations above 1.5 µg/mL, indicating a favorable therapeutic index.

Additionally, *Rhamnella gilgitica* leaf extract was used to biosynthesize γ-Fe_2_O_3_ NPs. These particles had an average size of ~21 nm, a polydispersity index of 0.737, and a zeta potential of −8.7 mV. The NPs were tested against *L. (L.) tropica* promastigotes and amastigotes (1-200 µg/mL, 72 h), with IC_50_ values of 9.63 µg/mL and 26.91 µg/mL, respectively. Biocompatibility assays showed CC_50_ values of 371.3 µg/mL for erythrocytes and 3548 µg/mL for macrophages [54], indicating low toxicity to mammalian cells and good safety margins.

Another formulation involved Fe_3_O_4_ SPIONs synthesized using coconut water. These particles had a spherical shape, average size of ~4 nm, and a zeta potential of −8.7 mV [55]. Although these NPs were effectively internalized by *L. (L.) amazonensis* promastigotes, they did not exhibit significant antiproliferative effects. However, cytotoxicity testing in RAW 264.7 macrophages revealed a high CC_50_ of 3420 µg/mL after 72 h, demonstrating high biocompatibility, though limited antileishmanial efficacy.

Finally, FeO NPs were biosynthesized using *Leptolyngbya* sp. L-2, a cyanobacterium belonging to the family Leptolyngbyaceae. The resulting NPs had an average size of ~23 nm, a polydispersity index (PDI) of 0.761, and a zeta potential of −8.5 mV, indicating a broad size distribution and a higher likelihood of aggregation. When tested against *L. (L.) tropica* promastigotes and amastigotes over a 72-hour period at concentrations ranging from 3.19 to 500 µg/mL, the FeO NPs exhibited IC_50_ values of 10.73 µg/mL and 16.89 µg/mL, respectively. Biocompatibility was assessed in human macrophages and erythrocytes, yielding CC_50_ values of 918.1 µg/mL and 2921 µg/mL, respectively [56], supporting the nanoparticles’ favorable safety profile and therapeutic potential. 

### Anti-*Leishmania* activity of iron oxide nanoparticles against axenic forms via hyperthermia-based approaches

The previously discussed studies primarily examined the effects of iron oxide nanoparticles (IONPs) applied alone to axenic forms of *Leishmania*. In contrast, some research has aimed to enhance the therapeutic efficacy of IONPs by combining them with hyperthermia techniques to improve parasite elimination. As shown in Figure 2, among the sixteen studies identified through searches in Science Direct, PubMed, and Scopus, only two investigated the use of IONPs in combination with hyperthermia.

One of these studies reported the green synthesis of ferromagnetic iron oxide nanorods (FIONs) using an aqueous extract of *Trigonella foenum-graecum* (fenugreek) seeds as a reducing and stabilizing agent. The resulting FIONs exhibited a polyhedral morphology and an average particle size of 70 nm. Characterization analyses revealed a polydispersity index (PDI) below 0.1 and a zeta potential of −23.4 ± 5.6 mV, suggesting a narrow size distribution and low tendency for aggregation [57]. These features are advantageous for biomedical applications, particularly in ensuring consistent cellular interactions and minimizing particle clustering in biological environments.

Following synthesis, the FIONs were applied in a photohyperthermia strategy, wherein infrared LED light irradiation is employed to heat the nanoparticles. This light-induced heating facilitates the conversion of absorbed energy into localized thermal energy, promoting the targeted destruction of *Leishmania* parasites. In this study, *L. (L.) tropica* promastigotes were exposed to the synthesized FIONs and subsequently irradiated with light emitted by an LED lamp rated at 84 lm/W. This exposure led to a significant temperature rise in the surrounding medium due to nanoparticle activation, resulting in high levels of parasite mortality. The treatment yielded an IC_50_ value of 0.036 ± 0.003 μg/mL, indicating a strong anti-parasitic effect. Mechanistic investigations attributed this efficacy to the induction of apoptosis and necrosis in the parasites, likely mediated by oxidative stress generated during the hyperthermic exposure (Figure 3).

In addition to evaluating efficacy, the study assessed the biocompatibility of the FIONs using both human erythrocytes and murine peritoneal macrophages. The NPs demonstrated high safety margins, with CC₅₀ values of 779 ± 21 μg/mL for human erythrocytes and 102.7 ± 2.9 µg/mL for mouse macrophages. These findings suggest that the FIONs exhibit minimal toxicity to host cells, reinforcing their potential for therapeutic application against *Leishmania* infections [57].

A second study utilized a distinct hyperthermia strategy - magnetic hyperthermia - to activate IONPs for parasite elimination. Unlike photothermal approaches, this technique employs an external alternating magnetic field to induce localized heating of the NPs through magnetic energy rather than light. In this study, commercially synthesized Fe_3_O_4_ NPs were produced using a conventional chemical co-precipitation method. To improve their stability and dispersion in aqueous media, the NPs were coated with citric acid (CA), yielding CA-coated Fe_3_O_4_ NPs. These NPs were spherical, with an average diameter of 33.1 nm. They exhibited a polydispersity index (PDI) of 0.20 and a zeta potential of −51.70 mV, reflecting good colloidal stability and a low tendency to aggregate [58].

To assess their anti-*Leishmania* activity, axenic amastigotes of *L. (L.) mexicana* were incubated with 200 µg/mL of the CA-coated Fe_3_O_4_ NPs. The infected cultures were then subjected to an alternating magnetic field (AMF) with an intensity of 30 mT and frequency of 452 kHz for a duration of 40 minutes. This treatment triggered nanoparticle heating and induced a significant reduction in parasite viability. Notably, confocal microscopy revealed a marked redistribution of the α-tubulin protein in the treated parasites, suggesting disruption of cytoskeletal architecture-a critical component of parasite viability (Figure 3).

### Anti-*Leishmania* activity of iron oxide nanoparticles against intracellular amastigotes

Although axenic cultures are useful for the initial screening of IONPs with anti-*Leishmania* activity, it is crucial to evaluate their effectiveness against intracellular amastigotes - the clinically relevant form of the parasite [5, 6, 7]. As shown in Figure 2, only four of the sixteen studies identified in our systematic search assessed the activity of IONPs against intracellular amastigotes. Notably, three studies were previously cited in this review for their effects on axenic forms. Their findings (summarized in Table 1) further highlight the therapeutic potential of IONPs in targeting *Leishmania* within host cells.

In one study, J774 macrophages infected with *L. (L.) major* were treated for 48 hours with Fe_3_O_4_ nanoparticles (NPs), either uncoated or coated with pyroctane olamine (PO), an antimicrobial and antifungal agent. Both formulations significantly reduced the rate of macrophage infection. The IC_50_ values were 31.3 ± 2.26 μg/mL for PO-coated Fe₃O₄ NPs and 62.3 ± 2.15 μg/mL for uncoated NPs. As shown in Figure 3, this reduction in infection correlated with plasma membrane damage in the parasites and increased production of nitric oxide (NO) [59], a well-established leishmanicidal molecule [39, 59]. NO exerts cytotoxic effects by targeting key parasite proteins such as glyceraldehyde-3-phosphate dehydrogenase (GAPDH), aconitase, and cysteine proteinases. Additionally, it promotes the formation of nitrosothiols (R-SNO) through reactions with thiol groups, thereby impairing protein function and synthesis in the parasite [24]. These findings suggest that IONPs exhibit both direct antiparasitic effects and immunomodulatory activity by enhancing macrophage-derived NO production.

Another study investigated the efficacy of MAA-functionalized Fe_3_O_4_@bio-MOFs against intracellular amastigotes of *L. (L.) major* in J774 macrophages. After 72 hours of exposure, a significant dose-dependent reduction in the number of intracellular amastigotes was observed. The negative control group averaged 5.5 amastigotes per macrophage, while treatment with 6.25, 25, 50, and 100 μg/mL of the nanomaterial reduced the counts to 2.8, 1.9, 1.4, and 0.95 parasites per macrophage, respectively [49]. These results highlight the high efficacy of MAA-functionalized Fe_3_O_4_@bio-MOFs in reducing intracellular parasitism.

Similarly, fenugreek-derived FIONs, when combined with 84 lm/W LED light, demonstrated potent activity against intracellular *L. (L.) tropica*. In this study, murine macrophages infected with the parasite were treated with the FIONs and light exposure, resulting in an IC₅₀ of 0.072 ± 0.001 μg/mL [57]. Photothermal activation likely enhanced the leishmanicidal effect through oxidative stress-induced apoptosis/necrosis.

Another study evaluated Fe_3_O_4_ SPIONs biosynthesized using coconut water for their activity against *L. (L.) amazonensis* in murine macrophages. Significant reductions in parasite load were observed at all tested concentrations (1-50 µg/mL) within 72 hours. Electron microscopy revealed morphological alterations in the amastigotes (Figure 3), such as lipid body formation, cytoplasmic disorganization, autophagic vacuoles, and parasitophorous vacuoles containing dead amastigotes and IONP aggregates [55]. These structural changes suggest multiple mechanisms of parasite damage.

Together, these studies demonstrate that IONPs, particularly when functionalized or combined with hyperthermic strategies, exhibit strong activity against intracellular amastigotes, supporting their potential use as nanotherapeutics for *Leishmania* infection.

### Anti-*Leishmania* activity of iron oxide nanoparticles against *in vivo* infection

*In vivo* studies are essential for evaluating the therapeutic potential of anti-*Leishmania* candidates. According to our systematic review, only three of the sixteen studies investigated the *in vivo* effects of IONPs (Figure 2). Notably, all three had previously demonstrated efficacy against axenic and intracellular forms of *Leishmania*, and their *in vivo* findings (summarized in Table 1) further support the potential of IONPs as alternative therapies for CL.

In one study, topical formulations of Fe_3_O_4_ NPs - both uncoated and coated with pyroctane olamine (PO) - significantly reduced skin lesion size in *L. (L.) major*-infected BALB/c mice [59]. Mice treated with uncoated Fe_3_O_4_ NPs showed average lesion size reductions of 4.8 mm and 6.1 mm at doses of 1 mg/kg and 2 mg/kg, respectively. In comparison, Fe_3_O_4_@PO NPs achieved greater reductions, with lesions shrinking by 8.1 mm and 9.0 mm at the same doses. A corresponding decrease in parasite burden was observed. Uncoated NPs reduced skin parasite load to 1.11 × 10³ and 0.81 × 10³ parasites, while PO-coated NPs reduced counts to 0.61 × 10³ and 0.39 × 10³, respectively. Untreated mice had an average parasite load of 2.66 × 10³. These results underscore the superior *in vivo* efficacy of PO-coated Fe_3_O_4_ NPs in reducing both lesion size and parasite burden in CL.

MAA-functionalized Fe_3_O_4_@bio-MOFs were also tested in a murine model of CL. In this study, the nanocomposites were incorporated into a Vaseline-based ointment at concentrations of 25 µg/mL and 12.5 µg/mL, with 0.1 mL topically applied three times per week. After 21 days, both concentrations significantly reduced lesion size and parasite burden in the spleen and liver of *L. (L.) major*-infected BALB/c mice (Figure 3) [49]. Immunological analysis revealed a marked increase in IFN-γ production by spleen lymphocytes in treated mice, while IL-4 levels remained low and statistically insignificant. These findings align with well-established models of adaptive immunity in CL, in which a Th1-type response - characterized by IFN-γ, TNF-α, IL-1β, IL-6, IL-12, IL-18, and IL-23 - is associated with parasite control and resistance [11, 13, 60-62]. Therefore, the data suggest that MAA-functionalized Fe_3_O_4_@bio-MOFs may exert therapeutic effects by promoting Th1-mediated immune responses.

Another study evaluated the therapeutic potential of PEI_25_-CAN-γ-Fe_2_O_3_ NPs in *L. (L.) major*-infected BALB/c mice using topical formulations in cream (0.02% and 0.067% w/w iron) and hydroxyethyl cellulose gel (0.067% w/w iron) [53]. These formulations significantly reduced lesion size and parasite load regardless of whether treatment was initiated 10- or 40-days post-infection. Remarkably, when treatment began at the time of infection, lesion development was entirely prevented. The formulations were effective at both high and low infectious doses and remained efficacious whether applied daily or every five days. According to the study, the antiparasitic effect was attributed to the cytolysis of *Leishmania* due to lysosomal disruption, likely caused by the polyethylenimine (PEI) coating on the NPs (Figure 3). These results highlight the promising therapeutic potential of PEI_25_-CAN-Fe_2_O_3_ NPs for CL treatment.

Collectively, these *in vivo* studies reinforce the therapeutic value of IONPs, particularly when surface-functionalized or combined with optimized delivery systems. Their demonstrated ability to reduce lesion size, decrease parasite burden, and modulate immune responses positions IONPs as strong candidates for the development of nano-based therapies to treat CL.

## Discussion

This review underscores that nanoparticles (NPs) composed of various forms of iron oxides have been extensively investigated for their anti-*Leishmania* activity. Most of these IONPs are spherical in shape, exhibiting significant variability in size (ranging from 5 to 90 nm), polydispersity index (0.2 to 1.0), and zeta potential (−13 mV to +35 mV). Despite these differences in morphological and dispersion characteristics, all IONPs consistently demonstrated antiparasitic effects, with IC_50_ values spanning from as low as 0.036 µg/mL to as high as 350.0 µg/mL. These findings provide compelling evidence that IONPs possess potent anti-*Leishmania* properties, positioning them as highly promising candidates for the development of novel therapeutic agents targeting CL.

The remarkable versatility of IONPs is also noteworthy, as they can be synthesized and functionalized through a wide array of approaches designed to enhance aqueous dispersibility, facilitate conjugation with other nanostructures for diverse biomedical applications, and enable the incorporation of antimicrobial agents to improve overall therapeutic outcomes. Application of IONPs across multiple experimental models of CL has yielded encouraging results, including a significant reduction in the viability of axenic promastigotes and amastigotes, decreased intracellular parasitism in infected macrophages, and diminished parasite burden within animal lesions. The studies reviewed suggest that IONPs exert their leishmanicidal activity primarily through direct parasiticidal effects, chiefly by disrupting cytoskeletal integrity and compromising intracellular organelles such as lysosomes following internalization by the parasite. Additionally, their antiparasitic activity is associated with the stimulation of nitric oxide (NO) production in infected macrophages, as well as the activation of pro-inflammatory cytokines, including interferon-gamma (IFN-γ), by lymphocytes. This dual mechanism - comprising both direct parasite killing and host immune system activation - underscores the therapeutic potential of IONPs as a novel strategy for controlling *Leishmania* infections.

The findings of this systematic review indicate that IONPs exhibit anti-*Leishmania* activity comparable to that of standard drugs currently used in the clinical management of CL. For instance, Glucantime® was effective against *L. (L.) major*, with an IC_50_ of 12.58 µg/mL after 72 hours of incubation [63], and also showed activity against intracellular amastigotes of *L. (L.) amazonensis* and *L. (V.) braziliensis*, with IC_50_ values of 22.9 µg/mL and 24.2 µg/mL, respectively [63]. Amphotericin B demonstrated activity against *L. (L.) tropica*, with IC_50_ values of 0.54 µg/mL for promastigotes and 0.60 µg/mL for axenic amastigotes [64] Similarly, miltefosine showed efficacy against intracellular amastigotes of *L. (L.) amazonensis*, *L. (V.) braziliensis*, and *L. (V.) guyanensis*, with IC_50_ values of 1.31 µg/mL, 2.20 µg/mL, and 1.64 µg/mL, respectively [65]. Collectively, the compiled data reinforce the therapeutic potential of IONPs, firmly positioning them as promising alternatives for the development of innovative and effective strategies to treat CL.

The biological activity and clinical applications of IONPs are influenced by multiple factors, including the synthesis method, which affects particle purity and reproducibility; particle size and morphology, which impact cellular uptake and biodistribution; surface charge and functionalization, which govern stability, targeting capabilities, and interactions with biological membranes; administration route and dosage, which determine bioavailability and therapeutic effectiveness; and interactions with host cells, including modulation of immune responses and potential cytotoxicity [66-69]. In this review, we found that IONPs evaluated for anti-*Leishmania* activity were synthesized via various methods and exhibited considerable diversity in shape, size, surface charge, dispersibility, and functionalization. Moreover, the assessment of their anti-*Leishmania* efficacy was conducted through a wide range of experimental protocols. This heterogeneity in nanoparticle characteristics and experimental designs complicates direct comparison between studies. Therefore, the establishment of standardized synthesis, characterization, and evaluation protocols is crucial to advance the field and facilitate the development of effective IONP-based therapies. 

Currently, only a limited number of iron oxide nanoparticle (IONP)-based products are approved for human use, primarily administered orally or parenterally. These formulations serve various clinical purposes, including iron supplementation for patients with renal disease, magnetic resonance imaging (MRI) contrast enhancement, and cell labeling applications [70]. However, IONPs can pose toxicological risks, including membrane disruption, mitochondrial dysfunction, oxidative stress via reactive oxygen species (ROS) generation, inflammation, DNA damage, and induction of apoptosis [67]. Some of these adverse effects were also reported in the reviewed studies, which identified poor biocompatibility of specific IONPs in certain experimental models. Systemically administered drugs, such as those delivered orally or parenterally, generally carry a greater risk of toxicity due to widespread distribution in the body, potentially affecting non-target tissues. In contrast, topical administration enables localized delivery, reducing systemic exposure and thereby lowering the likelihood of adverse effects. Notably, some studies included in this review reported IONPs with potent anti-*Leishmania* activity that may be suitable for topical formulation. This approach represents a promising strategy to minimize systemic toxicity while effectively targeting cutaneous lesions in the treatment of CL.

**Figure 4.**
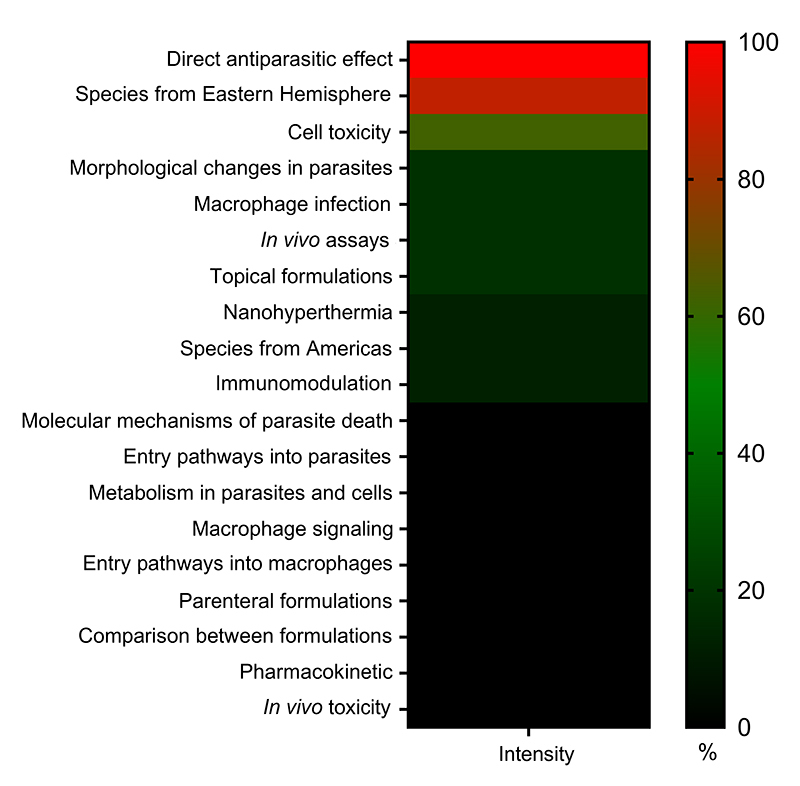
Heatmap illustrates the frequency of experimental parameters assessed across the sixteen studies included in this systematic review. Each article was evaluated for the presence of specific experimental approaches indicated in the heatmap. The number of studies assessing each parameter was expressed as a percentage of the total and ranked in descending order to emphasize the most commonly investigated parameters. The heatmap was generated using GraphPad Prism v10.0.

The findings reported in this review also indicated that most studies assessing the anti-*Leishmania* activity of IONPs have focused on species endemic to the Eastern Hemisphere. Further research is needed to investigate the effects of IONPs on species responsible for CL in the Americas (Figure 4 and Additional file 1). Future studies should aim to refine IONP formulations, assess their safety and efficacy in diverse animal models, and elucidate their mechanisms of action beyond the direct antiparasitic effects observed in axenic cultures. In addition to evaluating clinical potential, it is essential to explore the molecular and cellular pathways through which IONPs affect both the parasite and host target cells. Expanding knowledge on the pharmacodynamics and pharmacokinetics of IONPs during *in vivo* infection is critical to understanding their interactions within the host and identifying the most appropriate routes of administration. Moreover, long-term toxicity studies are necessary to fully characterize the safety profile of IONP-based therapies. These should include assessments of potential adverse effects on tissues and organs, as well as immunological responses. A comprehensive investigative approach will facilitate the development of safe and effective IONP-based treatments for CL, optimizing clinical outcomes while minimizing risks to patients.

This systematic review has certain limitations. First, it did not include a formal risk of bias or quality assessment of the included studies, which restricts the ability to fully evaluate their methodological rigor and reliability. Second, only articles published in English were included, potentially excluding relevant studies in other languages and introducing a risk of language bias. Additionally, the heterogeneity in experimental designs, evaluation methods, and reporting standards among the selected studies limited direct comparisons and hindered the ability to draw robust conclusions.

Despite the promising *in vitro* and *in vivo* results, translating IONPs into clinical settings for the treatment of CL still presents significant challenges. These include the need for standardized synthesis protocols to ensure reproducibility and scalability, comprehensive toxicological assessments to confirm long-term safety, and the optimization of delivery routes for effective targeting of infected tissues. Moreover, regulatory pathways for the approval of nanoparticle-based therapies remain complex and time-consuming, typically requiring extensive preclinical and clinical validation. While current evidence highlights the therapeutic potential of IONPs, further research is essential to overcome these barriers and advance their clinical application in the management of CL.

## Conclusion

IONPs have demonstrated substantial promise as potent anti-*Leishmania* agents, exhibiting significant parasiticidal activity across various experimental models of CL. Their unique physicochemical properties, versatility in synthesis and functionalization, and dual mode of action - combining direct parasite disruption with host immune activation - highlight their potential as innovative therapeutic candidates. While their efficacy appears comparable to conventional drugs, challenges such as variability in nanoparticle characteristics, limited standardization in experimental protocols, and concerns regarding biocompatibility and systemic toxicity must be addressed. Notably, topical administration emerges as a promising strategy to maximize local therapeutic effects while minimizing systemic risks. Future research should focus on optimizing IONPs formulations, expanding investigations to encompass diverse *Leishmania* species endemic to the American continent, and conducting comprehensive safety and pharmacokinetic evaluations. Overcoming these challenges through standardized methodologies and rigorous preclinical validation will be essential to translating IONPs into effective, safe clinical treatments for CL, ultimately advancing therapeutic options for this neglected tropical disease.

## Availability of data and materials

 All data generated or analyzed during this study are included in this article.

## Supplementary material

The following online material is available for this article:

Additional file 1. Experimental parameters considered for the heatmap construction. This table lists all parameters extracted from the included articles that were used to generate the heatmap presented in the article. For each parameter, the absolute number of studies in which it was assessed is provided, along with the corresponding percentage relative to the total number of articles included in the review.
